# Influence of garden cress seeds supplementation on immunity, hormonal status, and milk quality during the last third of pregnancy and lactation period of rabbit does

**DOI:** 10.1186/s12917-024-04381-5

**Published:** 2024-11-29

**Authors:** Yassmine M. El-Gindy, Soliman M. Zahran, Mohamed H. Ahmed, Azza Y. Idres, Sabrin A. Morshady

**Affiliations:** 1 Fish and Animal Production Department, Faculty of Agriculture (Saba Basha), Alexandria University, P.O. Box 21531, Alexandria, Egypt; 2 Animal Production Department, Faculty of Agriculture, Omar Al-Mukhtar University, Elbea, Libya

**Keywords:** Garden cress seed, Lactating rabbits, Immunity, Milk quality, Antioxidants

## Abstract

During the last third of pregnancy period, rabbits are exposed to many challenges, such as health complications and oxidative stress. The present study aims to use garden cress seeds (GAC) as a natural antioxidant to mitigate these challenges and evaluate its effects on reproductive performance, immunity, hormones, protein profile and milk quality. A total of 24 pregnant V-Line rabbits (pregnant at 20 days proved by palpation), with an initial body weight of 2395.83 g and about 6–7 months of age, were randomly distributed to 4 groups, the control group was fed the basal diet without GAC, the other treated groups GAC 3, GAC 4.5 and GAC 6 were fed the basal diet supplemented with 3, 4.5, and 6% GAC, respectively. The experiment lasted six weeks. The results revealed that treated female rabbits with different levels of GAC did not have a noticeable effect on milk yield, average daily milk yield, or most of the milk analysis parameters (milk density, total solids, solids not fat, lactose, ash, and protein). On the other hand, GAC showed a significant increase in milk fat, and GAC 3% rabbits displayed a higher percentage of milk fat (*p* < 0.050) than the other groups. Likewise, treatment with GAC also had a significant impact on serum total protein, globulin, IgG, and IgM. The 3% GAC rabbits showed significantly higher total protein, globulin, and IgM values than the control group. However, all GAC treatments significantly improved serum IgG and the best value was found in the 3% GAC group. In conclusion, our results demonstrated that feeding lactating rabbits a diet supplemented with GAC could be considered a good strategy to enhance immunity, protein profile, MY and milk fat. The best results were obtained with the GAC 3% treatment.

## Introduction

With the rapid expansion of the rabbit breeding industry, the rabbit sector, especially in Egypt and generally in the world, is facing serious challenges to sustainable development and the growing demand for animal protein [1, 2]. One of these challenges is the stressful periods, represented by pregnancy and lactation, that cause animals to suffer from oxidative stress [3]. Oxidative stress and high levels of reactive oxygen species (ROS) can damage cells, reducing their normal biological activity, including their energy metabolism, and disrupting cell signal transduction, that can lead to damage to proteins, lipids, DNA, and enzymes [4].

Antioxidants play an important role as a defense strategy of the animal body and help to deal with oxidative stress caused by ROS during stressful periods such as pregnancy and lactation period. Plant seeds are important sources of antioxidants, and there is increasing interest in the antioxidant analysis of plants [5]. One of the seeds of plants that has a principal source of potent therapeutic drugs and has caught the attention is garden cress [6]. The GAC, as a natural antioxidant, offers advantages as it is readily available, inexpensive, does not have any side effects [7], and can be grown in any type of climate or soil [8].

Garden cress (*Lepidium sativum L.*) is a common herb widely spread throughout the world, used as a therapeutic and nutritional agent and has many biological and health-promoting activities in animals [9]. The garden cress seeds are brownish red in color, small, pointed, oval-shaped, triangular at one end, smooth, about 3–4 mm long and 1–2 mm wide [7, 10]. Garden cress seed contains a wide range of bioactive components, as shown in Table 1, including flavonoids, mainly apigenin, quercetin, kaempferitrin [11], kaempferol, glucuronide, gallic acid, protocatechuic acid, coumaric acid, caffeic acid, terpenes, glucosinolates, and many more [12].

**Table 1:** Bioactive compounds of garden cress seeds

Bioactive compounds	Amount (µg/100 g)
Gallic acid	3001.75
Coumaric acid	517.52
Caffeic acid	212.55
Kaempferol	58.05
Quercitrin	1520.33
Protocatechuic acid	582.23

Source (Tufail et al., 2024)

Pharmacological evaluation suggested that GAC seeds are generally applied in the healing of many clinical disorders, including hepatitis, sexual debility, vitamin C deficiency, constipation, and migraine [13]. The seeds of garden cress are considered galactagogue, emmenagogue, and are laxative and used as a dressing for sores in camels and horses [14]. It was also used to prepare a solution to massage infected cow udders [15]. The hypothesis was to investigate and clarify how GAC supplements affect female lactation. The purpose of this study is to determine the lactation performance, immunity and hormonal profiles of lactating rabbits. Also, evaluate the possible beneficial effect of antioxidants on the blood protein profile and immunoglobulin.

## Materials and methods

### Source of garden cress seed

The seeds of garden cress were obtained from a local market (Fathala Gomlla Hypermarket, Alexandria Governorate, Egypt). Then it was washed and dried, then rushed to fine powder by a mixer equipped with a stainless-steel blade and stored in an airtight container.

### Experimental diets and animals

In this study, a total of 24 pregnant V-Line rabbits (pregnant at 20 days proven by palpation), with an initial body weight of 2395.83 g and about 6–7 months of age, were used. The rabbits were obtained from a research farm of the Faculty of Agriculture of Alexandria University, Egypt. To comply with the 3R protocol, the minimum number of lactation rabbits needed to test the hypothesis without affecting statistical power was used [16, 17]. All rabbits were housed at the Rabbit Production Lab, Faculty of Agriculture, Saba Basha, Alexandria University, Egypt. The Lab. temperature was 19–24 °C; humidity was 40–64% with 16 h light and 8 h dark. All animals received a seven-day acclimatization period to the laboratory environment before the experiment began. The basic ingredients of the diet presented in Table 2 covered the nutritional requirements of rabbits [18].

**Table 2:** Ingredients of rabbit’s basal diet

Ingredients (g/ kg)	Control
Maize grain	160.00
Barley	70.00
Wheat bran	190.00
Soya bean meal 44	200.00
Clover hay	240.00
Wheat straw	100.00
Beet molasses	20.00
Premix^1^	3.00
Calcium Carbonate	2.00
Di-Calcium Phosphate	8.00
Salt (NaCl)	5.00
L-Lysine HCL − 98%	1.50
Methionine - DL − 99%	0.50
Garden cress	0.00

Vaccinations against the main diseases were planned. The pregnant rabbits were randomly divided into four groups, six rabbits per group, each female as individual replicates. The control group was fed the basal diet without GAC, and the GAC 3, GAC 4.5 and GAC 6 groups were fed the basal diet supplemented with 3, 4.5 and 6% GAC, respectively. According to [19], the selected levels of GAC seeds are at safe levels without any compromising the health status or growth performance in V-Line rabbits. The different levels of GAC were mixed with the basal diet ingredients before pelleting the experiment diets.

All experimental rabbits were housed individually in metallic cages under hygienic laboratory conditions and had facilities for feeding and watering. The diet and clean drinking water were offered *ad libitum* to all the rabbits throughout the experiment. The experiment lasted 42 days. Litter weight, size, and bunny weight were recorded to calculate the average of each of them.

### Milk yield and analysis

The milk yield (MY, g) of all experimental rabbit does was measured once a week after giving birth, at 7:00 am. To do this, the rabbit was weighed before and after suckling using a digital scale. The bunnies were separated from their mothers for 24 h prior to measurement [20]. The daily MY of each week was multiplied by 7 and then added together to get the total MY.

The average daily milk yield was calculated by dividing the sum of the weekly milk yield for each female by 4. Milk samples (15–20 mL) were collected twice in 1st and 3rd weeks by gently massaging the mammary glands after injection of females with 3–5 IU oxytocin into the marginal ear vein to stimulate milk ejection then put into sterilized plastic bottles and stored at -20 C until further analysis. The milk was analyzed for total solids, fat, protein and ash using a Milko Scan FT 6000, Foss Electric, Hillerod, Denmark. Lactose was determined by subtracting the sum of fat, protein, and ash from the total solids.

Milk immunoglobulin IgA concentration was measured on the 1st week of lactation by Sandwich ELISA, double antibody using a quantitative commercial kit (Assay Genie Company) of Rabbit IgA ELISA (RBFI00052). The milk yield coefficient (M) was calculated according to the method described by [21] with the following formula:

$${\rm{M}}\, = \,\left[ {\left( {{\rm{LW}}2{\rm{ }} - {\rm{ LW}}1} \right):{\rm{ }}\left( {21{\rm{ }} \times {\rm{ LW}}2} \right)} \right]\, \times \,100$$

where: LW1 - litter weight (g) on day 2 postpartum, LW2 - litter weight (g) on day 21 postpartum.

### Blood collection and biochemical analysis

Blood samples were collected from five rabbits from each group on the 21st day of lactation after an overnight fasting. Blood samples were collected from the marginal ear vein in vacuum tubes without anticoagulant. After the supernatant was separated, the blood was centrifuged (700 r/min). The serum was transferred to clean 1.5 ml tubes and stored frozen at -20 ° C until analysis.

Total protein and albumin concentrations were determined using commercial kits (Biodiagnostic Co., Cairo, Egypt). Globulin was calculated by subtracting albumin from total protein. Thiobarbituric acid reactive substances (TBARS) was measured according to [22]. The immunoglobulin IgG and IgM concentrations were measured using a quantitative commercial kit (Kamiya Biomedical Company) of Rabbit IgM ELISA (KAM-KT-614) and IgG ELISA (KAM-KT-517).

The hormones assay (prolactin and oxytocin) was determined using the Rabbit ELISA Kit according to the manufacturer’s instructions (MyBiosource, Inc, San Diego, CA 92195-338, USA), with a Microplate ELISA reader (Mindary, MR-96 A, China).

### Statistical analysis

All original data were checked for normal distribution and the homogeneity of the data, and it was found that all data followed the normal distribution. The statistical analysis for the GAC treatments was performed by using the general linear model (GLM). Polynomial contrasts were used to test the linear, quadratic, and cubic effects of increasing level of GAC supplementation. One-way analysis of variance (ANOVA) followed by Tukey’s test was used for multiple comparisons between groups to identify significant differences at *p ≤* 0.05 (IBM SPSS Statistics for Windows, Version 20, Chicago, IL, USA). After testing the normal distribution and homogeneity of the data, it was found that all data follow the normal distribution. The figures were fitted by SigmaPlot 14.0 (Systat Software Inc.).

## Results

### Reproductive performance

The current results in Table 3 demonstrated a non-significant influence of GAC treatments on studied reproductive performance parameters (litter size, litter gain and bunny weight gain).

**Table 3:** Impact of dietary garden cress seed levels in some reproductive performance of lactating female rabbits

	Control	Garden cress seeds %			SEM	*P*- value			
		3	4.5	6		Treat	Liner	quadratic	Cubic
Aver. Litter size (no.)	5.95	7.83	6.56	5.78	0.37	0.144	0.586	0.586	0.221
Aver. Litter gain (g.)	713.75	1185.83	1026.00	723.25	106.07	0.313	0.897	0.085	0.587
Bunny weight gain (g.)	255.69	283.13	246.52	234.84	12.38	0.555	0.429	0.459	0.422

### Milk yield and milk analysis

**Fig. 1:**
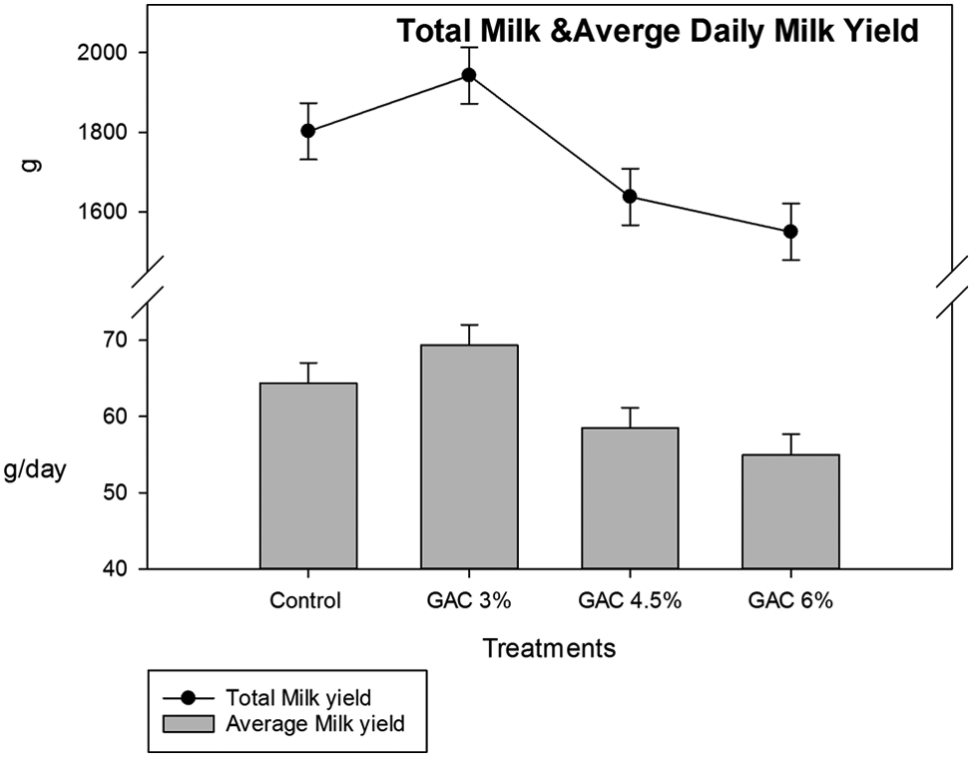
Impact of dietary garden cress seed (GAC) levels in total milk and average daily milk yield of lactating female rabbits

**Fig. 2:**
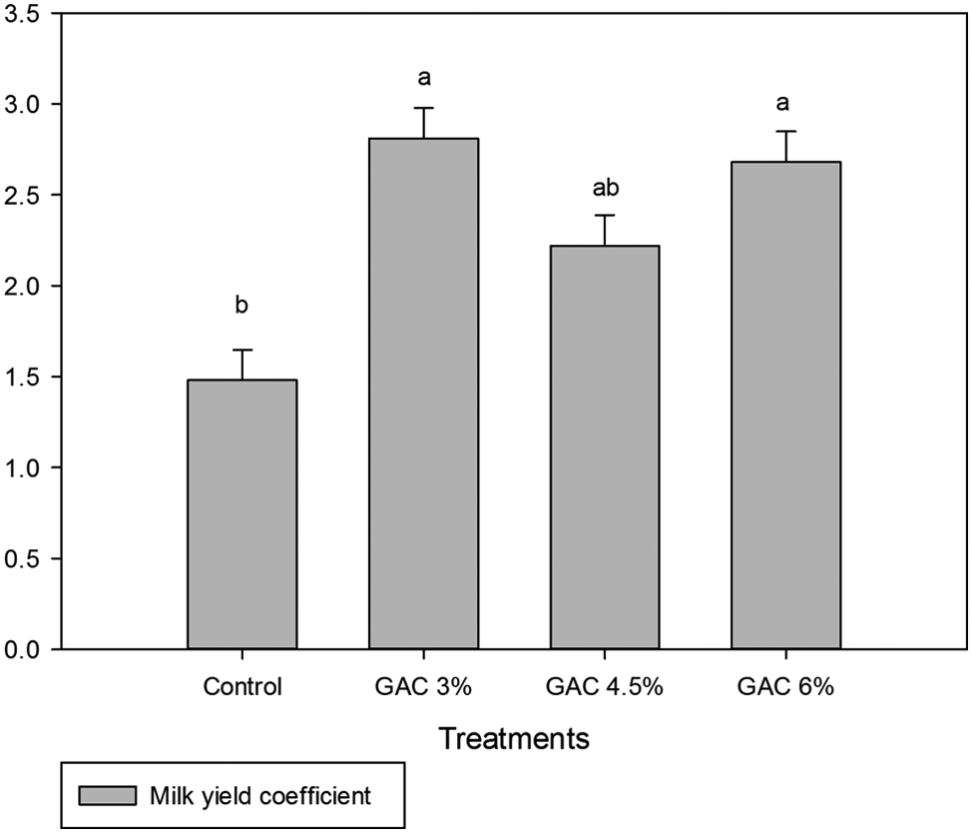
Impact of dietary garden cress seed (GAC) levels in milk yield coefficient of lactating female rabbits. Means in column with different superscript letters (a–b) are significantly different (*p ≤* 0.05)

The results of total milk yield and average daily milk yield as affected by dietary GAC supplementation are shown in Fig. 1. Treatment of female rabbits with different levels of GAC did not have a noticeable effect on MY and average daily milk yield. While in Fig. 2 the results revealed that dietary supplementation of GAC at different levels significantly improved the milk yield coefficient, doe rabbits in the GAC 3% and GAC 6% groups significantly recorded a higher milk yield coefficient than in the control group.

**Fig. 3:**
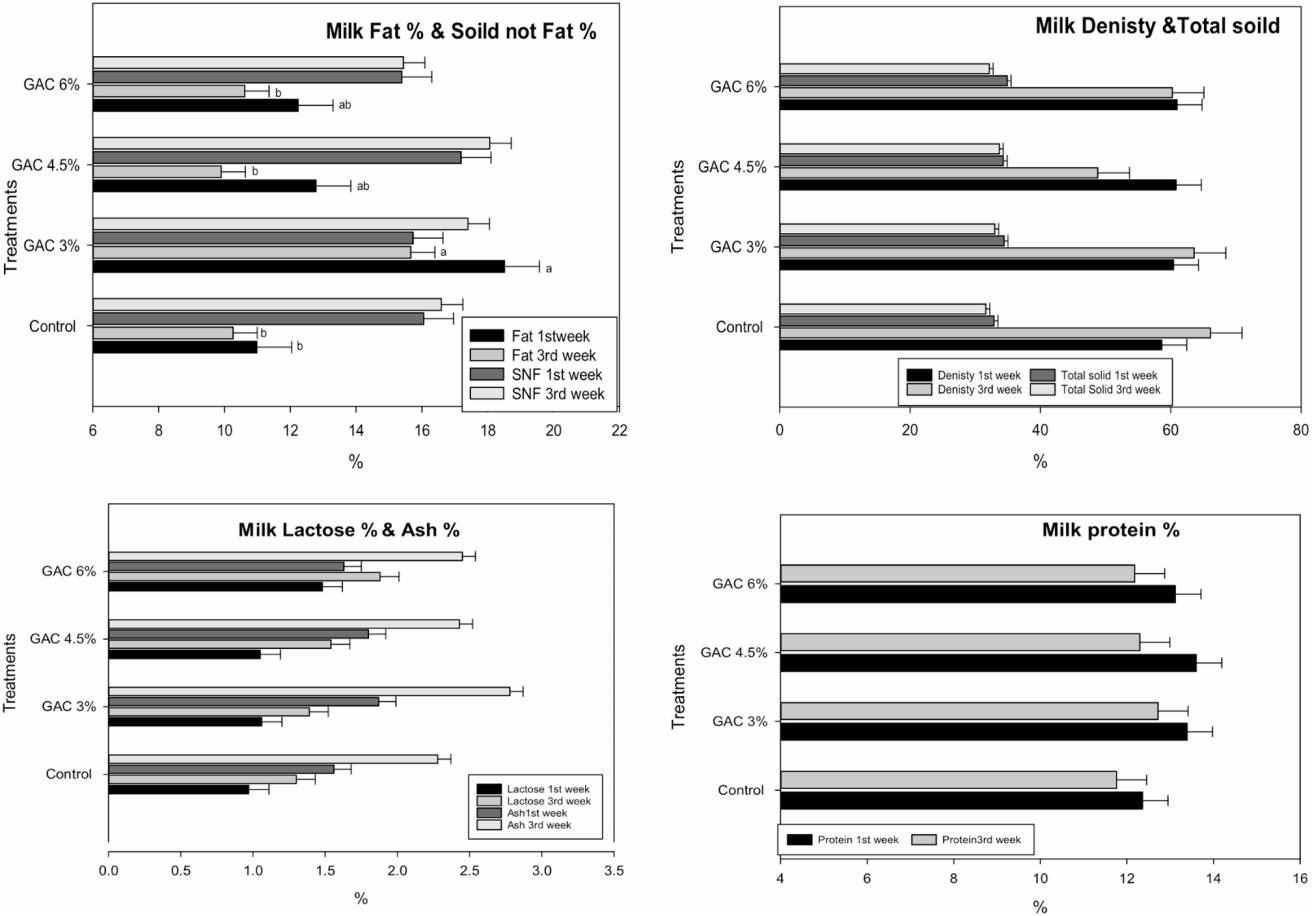
Impact of dietary garden cress seed (GAC) levels in Milk analysis profile of lactating female rabbits. Means in column with different superscript letters (a–b) are significantly different (*p ≤* 0.05)

The milk analysis of the lactation rabbit as influenced by GAC treatments is reported in Fig. 3. Dietary GAC at different levels did not affect (*p >* 0.050) most of the milk analysis parameters (milk density, total solid, solid not fat, lactose, ash, and protein). Whereas GAC had a significant effect on milk fat and GAC 3% showed a significant (*p* ≤ 0.050) increase in percentage of milk fat compared to other groups.

**Fig. 4:**
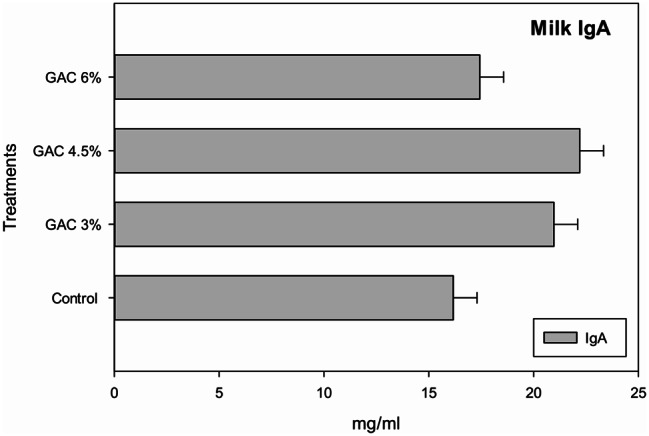
Impact of dietary garden cress seed (GAC) levels in milk imumnoglobuline A of lactating female rabbits

Milk immunoglobulin A showed a non-significant improvement in the 3% and 4.5% groups compared to the other groups (Fig. 4).

### Biochemical blood

The protein profile of lactating rabbit does as affect by GAC supplementation is shown in Table 4. The results revealed that GAC treatments had a significant impact on serum total protein, globulin, IgG, and IgM. The total protein, globulin and IgM values of the rabbits in the GAC 3% group were considerably higher than those of the control group; the difference between the GAC 3% and GAC 4.5% groups was not statistically significant.

**Table 4:** Impact of dietary garden cress seed levels in serum protein profile and immunoglobulin (ig) concentration of lactating female rabbits

	Control	Garden cress seeds %			SEM	*P*- value			
		3	4.5	6		Treat	Liner	quadratic	Cubic
**Protein Profile**:									
T. Protein (mg/dl)	5.59^b^	7.10^a^	6.30^ab^	5.43^b^	0.22	0.007	0.353	0.002	0.122
Albumin (mg/dl)	3.45	3.80	3.71	3.43	0.12	0.666	0.892	0.238	0.833
Globulin (mg/dl)	2.14^b^	3.30^a^	2.59^ab^	2.00^b^	0.18	0.020	0.361	0.006	0.125
Albumin/ Globulin	1.64	1.24	1.46	1.84	0.12	0.349	0.434	0.121	0.670
**Immunity**									
IgG (mg/dl)	915.48^d^	994.99^a^	963.40^b^	936.72^c^	7.72	0.001	0.001	0.008	0.944
IgM (mg/dl)	49.48^b^	52.07^a^	51.63^ab^	49.71^b^	0.38	0.012	0.003	0.563	0.126

Means in row with different superscript letters (a–c) are significantly different (*p* ≤ 0.05)

All GAC treatments significantly improved serum IgG concentration and the best value was found in the GAC 3% group. Both albumin and the ratio between albumin / globulin did not change due to GAC treatments.

**Fig. 5:**
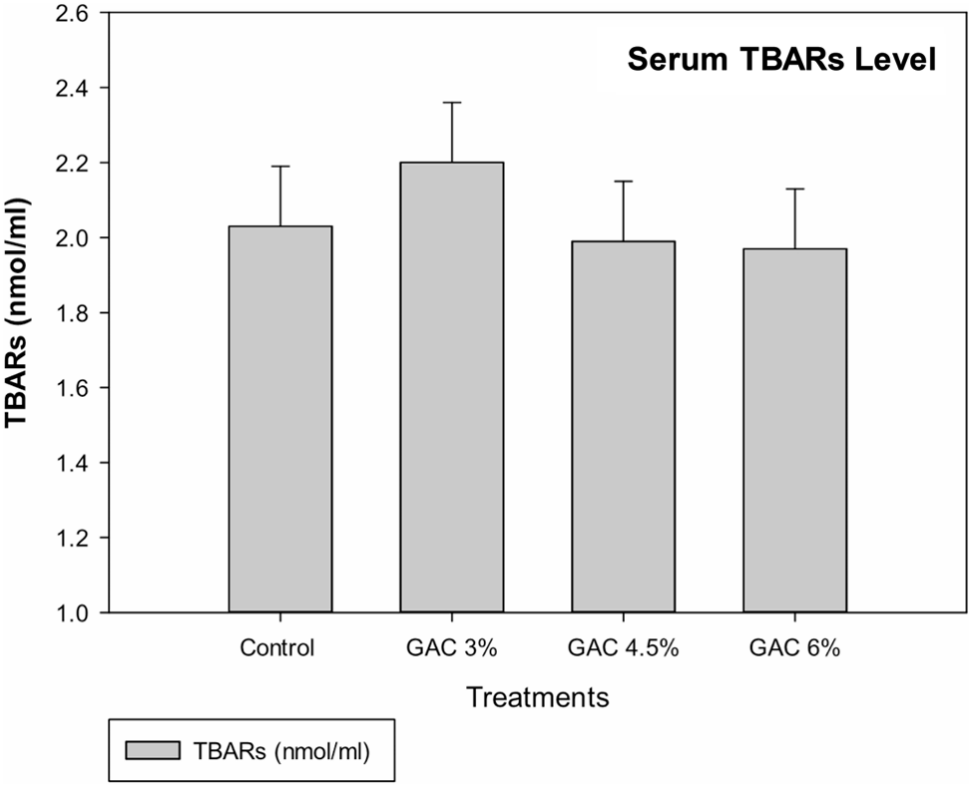
Impact of dietary garden cress seed (GAC) levels in blood TBARs of lactating female rabbits

**Fig. 6:**
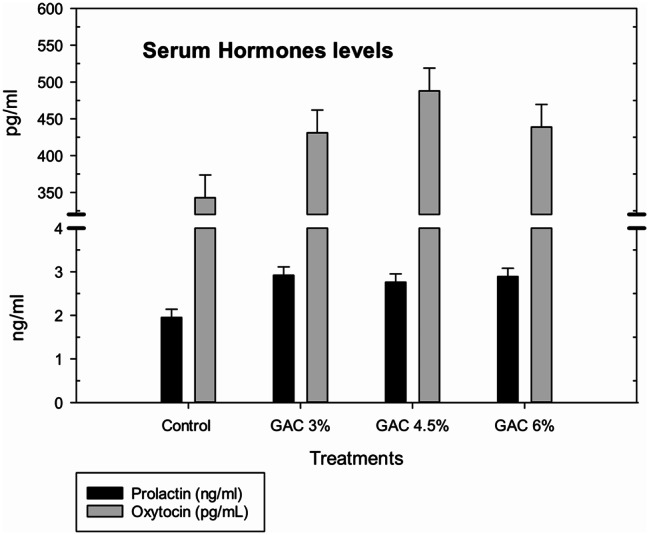
Impact of dietary garden cress seed (GAC) levels in blood hormones (prolactin and oxytocin) of lactating female rabbits

The blood oxidation and hormone levels of lactation rabbits affected by dietary supplemented with GAC are shown in Figs. (5&6). The results showed that GAC treatments did not have a noticeable effect on the level of blood TBARs as an oxidation marker and tended to increase the concentration of measuring hormones (prolactin and oxytocin). The GAC 3% rabbits insignificantly recorded the highest blood prolactin level while; GAC 4.5% achieved the best value of blood oxytocin level.

## Discussion

The findings of this study did not show significant differences between the GAC treated groups and the control for the reproductive performance parameters studied (average of litter size, litter gain, and bunny weight gain). These findings are confirmed by the previous study conducted by [23] that found no significant differences in litter size between all experimental groups of GAC on the third week of lactation. Our results are in line with [24] which observed no significant impact on litter size among the 5, 7 and 10% of the GAC treated groups. Similaraly [25] found that the total body weight gain and the average daily gain of rabbits did not differ significantly between the 5 and 7.5% of the GAC treated groups.

Although the results of the present study are not consistent with the [26] that confirm the ability of GAC herbs to improve reproductive performance and fertility through phytochemicals by increasing levels of reproductive hormones and improving the antioxidant state and reproductive performance by improving the hypothalamic-pituitary-gonadal axis. This may be due to the short extension period of the present study.

A non-significant difference in total and average milk yield could be the reason for the non-significant difference in the litter size due to the positive relation between MY and litter size [27].

There was a significant improvement in the milk yield coefficient and milk fat percentage by feeding lactated rabbits with GAC compared to the control. Our results agree with [7] that found garden cress can be used in a diet as a nutritional supplement for lactating women to increase of milk secretion during the postnatal period. In addition, GAC having chemicals such as estrogen help to regulate and stimulate milk production [28].

The significant increase in milk fat in all GAC treatments may be due to the supplies body-building proteins and energy-giving milk fat [29], which may be due to the positive relationship between the dietary cress seeds and improvement in the digestibility of ether extract and the nitrogen balance [23].

There was a significant improvement in serum total protein, globulin, IgG, and IgM by using GAC. The increase in blood protein profile in animals fed a diet enriched with GAC may be due to the hepatoprotective effect of GAC by own-regulated pro-inflammatory cytokines (e.g. TNFα and IL-6 mRNA), stress genes (iNOS and HO-1) and increased IL-10 expression of IL-10 dose-dependently [30]. Furthermore, pretreatment with extract leads to downregulation of nuclear NF-κB (p65), NF-κB-DNA binding activity, myeloperoxidase (MPO) activity, and nitric oxide level [31], or by reducing oxidative stress, inflammation, and apoptosis in the liver [32].

Consistent with our findings [4, 33], found that there were significant increases in mice’s immune system and general health by feeding GAC extract. Furthermore, treating diabetic rats with cress seed significantly increased immunoglobulins (IgG, IgA and IgM) compared to the control group [34]. Furthermore, increasing dietary levels of GAC increased the immune status of growing rabbits by increasing SRBC levels and the highest significant levels were observed after 14 days of treatment. Therefore, these findings suggest that the GAC diets enhance the immune system by increasing blood IgG and IgM levels or by having acids like arachidic acid, folic acid, ascorbic acid, and beta carotene which help to improve the immune system [7]. Additionally, garden cress seeds contain Vitamin C and Vitamin A, which have good immune stimulating activity. Vitamin C improves the function of white blood cells and is characterized by antibacterial and antiviral activity. Vitamin A helps prevent viruses and bacteria from penetrating the body through sensitive mucous membranes in the eyes, mouth, nose, throat, lungs, and stomach [35].

Thiobarbituric acid reactive substances (TBARS) are an indicator of oxidative stress in biological systems and the level of lipid peroxidation in tissue [36], GAC treatments did not have a noticeable effect on blood TBARs and hormones (prolactin and oxytocin) in the present study. Although some research confirmed that GAC seeds have radical scavenger and antioxidant activities due to total phenolic content, our data get al.ong with [37, 38] that confirm that blood antioxidants and TBARS were not significantly affected by GAS supplementation.

## Conclusion

Improved immunity, blood protein levels, milk yield coefficient, and milk fat content were observed in doe rabbits fed garden cress seeds (*Lepidium sativum*) during the last third of pregnancy and lactation. This suggests that incorporating 3% garden cress seeds into rabbit does diets can enhance overall maternal and offspring well-being.

## Data Availability

The data used to confirm our study’s findings are included in the article, and data coding is available from the corresponding author upon reasonable request.
